# Ligature of the Common Carotid Artery, and Experiments on the Ligature of Both Carotids in Animals

**Published:** 1841-08

**Authors:** 


*Ligature of the common Carotid Artery, and Experiments on the Ligature of both Carotids in Animals.*—M. Jobert, de Lamballe, has addressed a memoir to the Royal Academy of Medicine, containing a case where he tied the common carotid to cure an erectile tumour of the orbit, and also the result of his experiments on living animals, to determine the influence that ligature of both carotids would have upon them.

Ligature of the common carotid on either side does not stop the course of the blood through the ramifications of the tied artery; how then can it effect the cure of erectile tumours of the face and head? M. Jobert replies, 1st, by the sudden subtraction of a large quantity of blood from the tumour; 2d, by giving an obstacle to the transmission of the impulse of the heart, in its full energy towards the tumour. He has convinced himself by his experiments, that beyond the ligature, the blood runs in a continual jet, waving, and without jerk (*saccade.*) His conclusions are, 1st, that erectile tumours of the orbit, destroy the bones after the manner of aneurism; 2d, they have the characters of aneurismal tumours, and are cured by ligature of the common carotid of the same side; 3d, the cure is not owing to obliteration of the artery beyond the ligature, but to diminish all impulse of the column of blood arriving in the tumour; 4th, the vertebral arteries suffice for the cerebral circulation after ligature of the carotids; 5th, dogs, sheep, and rabbits do not experience ill effects after this operation; 6th, horses on the contrary do not survive it, dying with pulmonary apoplexies; 7th, blood-lettings before or after the ligature, diminish the intensity of the pulmonary lesions; 8th, perhaps in man, the loss of a certain quantity of blood after the operation, would have good effect.—*Rev. Med. Sept.* 1840.

MM. Berard, Gimelle, and Larrey, who were appointed as a committee to examine the memoir of M. Jobert, consider it of importance; 1st, as adding two cases of cure of erectile tumours, one in the orbit by ligature of this carotid, to four other cases previously recorded; 2d, as the experiments prove the harmlessness of ligature of both carotids, in those animals whose vertebral arteries enter the cranium of a calibre sufficiently large to keep up the cerebral circulation and avoid pulmonary congestion; 3d, as it proves that the brain and the organs of the senses preserve their functions entire after this ligature; 4th, as it places beyond doubt, that animals who from their anatomical construction survive the ligature of the common carotid arteries, are not affected with lesions of the organs of the senses as described by authors; 5th, as these experiments and their results will exercise a great influence on the surgical therapeutics of diseases, for the cure of which ligature of the common carotids might be proposed.—*Ibid.*, from *Bull. de l' Acad. Royale de Med.*, Oct. 15 and 30, 1840.

